# Synergistic In Vitro Antiviral Effect of Combinations of Ivermectin, Essential Oils, and 18-(Phthalimid-2-yl)ferruginol against Arboviruses and Herpesvirus

**DOI:** 10.3390/ph16111602

**Published:** 2023-11-13

**Authors:** Liliana Betancur-Galvis, Orlando José Jimenez-Jarava, Fatima Rivas, William E. Mendoza-Hernández, Miguel A. González-Cardenete

**Affiliations:** 1 Grupo GRID—Grupo de Investigaciones Dermatológicas, Instituto de Investigaciones Médicas, Facultad de Medicina, Universidad de Antioquia, Medellín 050010, Colombia; ojose.jimenez@udea.edu.co; 2 Department of Chemistry, Louisiana State University, 133 Chopping Hall, Baton Rouge, LA 70803, USA; frivas@lsu.edu; 3 Instituto de Tecnología Química, Universitat Politècnica de València-Consejo Superior de Investigaciones Científicas, Avda. de los Naranjos s/n, 46022 Valencia, Spain; wemenher@itq.upv.es

**Keywords:** ivermectin, ribavirin, acyclovir, ferruginol, direct-acting antivirals, host-targeting antivirals, drug combinations, CHIKV, ZIKV, HHV-2

## Abstract

Combining antiviral drugs with different mechanisms of action can help prevent the development of resistance by attacking the infectious agent through multiple pathways. Additionally, by using faster and more economical screening methods, effective synergistic drug candidates can be rapidly identified, facilitating faster paths to clinical testing. In this work, a rapid method was standardized to identify possible synergisms from drug combinations. We analyzed the possible reduction in the antiviral effective concentration of drugs already approved by the FDA, such as ivermectin (IVM), ribavirin (RIBA), and acyclovir (ACV) against Zika virus (ZIKV), Chikungunya virus (CHIKV), and herpes virus type 2 (HHV-2). Essential oils (EOs) were also included in the study since they have been reported for more than a couple of decades to have broad-spectrum antiviral activity. We also continued studying the antiviral properties of one of our patented molecules with broad-spectrum antiviral activity, the ferruginol analog 18-(phthalimid-2-yl)ferruginol (phthFGL), which presented an IC_99_ of 25.6 μM for the three types of virus. In general, the combination of IVM, phthFGL, and oregano EO showed the greatest synergism potential against CHIKV, ZIKV, and HHV-2. For instance, this combination achieved reductions in the IC_99_ value of each component up to ~8-, ~27-, and ~12-fold for CHIKV, respectively. The ternary combination of RIBA, phthFGL, and oregano EO was slightly more efficient than the binary combination RIBA/phthFGL but much less efficient than IVM, phthFGL, and oregano EO, which indicates that IVM could contribute more to the differentiation of cell targets (for example via the inhibition of the host heterodimeric importin IMP α/β1 complex) than ribavirin. Statistical analysis showed significant differences among the combination groups tested, especially in the HHV-2 and CHIKV models, with *p* = 0.0098. Additionally, phthFGL showed a good pharmacokinetic profile that should encourage future optimization studies.

## 1. Introduction

Ivermectin (IVM) is a semisynthetic mixture of two avermectin B derivatives consisting of a large macrocyclic lactone ring originally produced by the soil actinomycete, *Streptomyces avermectinius* [1]. IVM is a broad-spectrum anti-parasitic drug approved by the FDA that has also demonstrated in vitro antiviral activity against a number of DNA and RNA viruses, including severe acute respiratory syndrome coronavirus 2 (SARS-CoV-2) [2]. A systematic review published in *The Journal of Antibiotics* summarized the antiviral effects of IVM by reviewing available in vivo and in vitro studies over the past 50 years [3]. IVM exhibits antiviral activity in a variety of virus genera, such as *Alphavirus*, *Alphainfluenzavirus*, *Arterivirus*, *Betacoronavirus*, *Circovirus*, *Flavivirus*, *Lentivirus*, *Polyomavirus*, and *Varicellovirus*, and its main mechanism of action is the inhibition of the host heterodimeric importin (IMP) α/β1 complex, responsible for the nuclear accumulation of some viral proteins [3,4,5,6].

Essential oils (EOs) are very important natural products obtained from aromatic plants containing two main groups of biosynthetic constituents: terpenes/terpenoids and aromatic/aliphatic constituents, which are further classified based on their molecular weight [7]. Their extended use in many industries, including pharmaceuticals, agriculture, perfumes, and food, is predominantly attributed to their distinctive aromas and intriguing bioactivities [7,8]. EOs are composed principally of volatile substances containing complex mixtures of organic compounds that exhibit a wide range of promising biological activities, including antibacterial, antifungal, antiviral, antispasmodic, antioxidant, antiparasitic, anthelmintic, insecticidal, anti-inflammatory, and cytotoxic activities [8]. There are approximately 3000 EOs obtained from different plant species, of which only 300 are economically important for the perfume, food, agricultural, sanitary, and pharmaceutical industries, due to their characteristic fragrances and interesting pharmacological actions [8]. Of particular interest are some EOs that exhibit antiviral activity against acyclovir-resistant strains of human herpesvirus (HHV), with activity up to 100 times greater than that of acyclovir [9]. There are numerous publications on the anti-herpetic activity of EOs and their isolated active compounds [10,11], but EOs also have potential as broad-spectrum antivirals against other DNA- and RNA-enveloped viruses [8]. Their mechanism of action primarily involves altering the viral envelope through interactions with viral structural proteins, disrupting the early stages of viral replication such as cell entry and adsorption [12,13]. Recent studies have also documented that EOs can target cellular processes, leading to differential expression of interferon-response-related genes [14,15].

**Figure 1:**
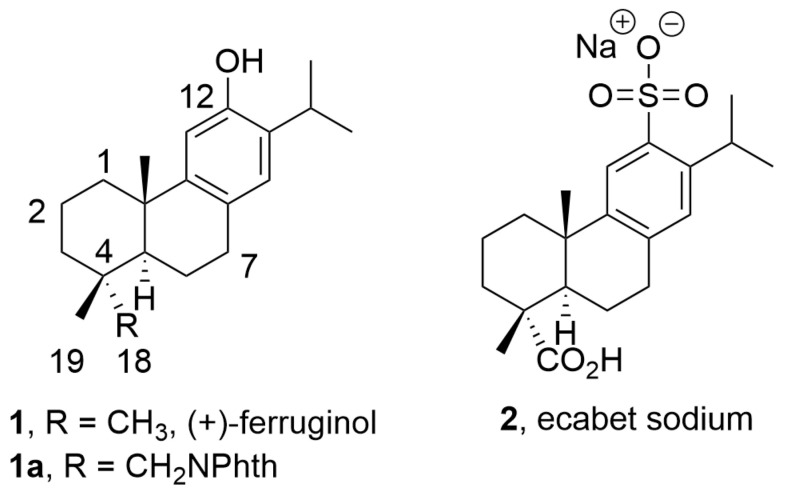
Chemical structure of bioactive abietanes.

During our compound screening program designed to search for new antivirals derived from natural products, we discovered a broad-spectrum antiviral analog of the bioactive abietane-type diterpene ferruginol (**1**, Figure 1) [16,17], which we later named 12-hydroxy-N,N-phthaloyldehydroabietylamine or 18-(phthalimid-2-yl)ferruginol (**1a**, henceforth abbreviated as phthFGL, Figure 1) based on the abietane carbon skeleton numbering (Figure 1) [18,19,20,21]. PhthFGL (**1a**) has exhibited relevant in vitro antiviral activity against herpes and dengue [18], Brazilian Zika strains [19], Colombian Zika and chikungunya strains [20], and very recently, against the human coronavirus 229E (HCoV-229E) [21]. With the aim of gaining insight into the possible mechanism of action of phthFGL, we previously carried out a basic computational study using a molecular docking approach [20]. In that work, it was found that phthFGL has a good affinity for key viral targets, including chikungunya nonstructural protein 2 (nsP2), Zika and dengue virus NS5 methyltransferase, and herpesvirus thymidine kinase (TK) with a higher binding energy value than that of acyclovir itself [20]. Additionally, like IVM, phthFGL showed features suggesting potential inhibition towards cellular therapeutic targets, as demonstrated by its high free energy value with G-Actin and tubulin [20,22]. However, experimental evidence to confirm those predictions needs to be obtained, though some evidence gathered by collaborators points to this molecule as a host-targeted antiviral [22]. PhthFGL is structurally an analog of ferruginol (**1**), a well-known bioactive molecule, which possesses a phthalimide moiety attached at the C18 position. Semisynthetic bioactive abietane-type compounds are readily obtained from renewable commercial starting materials such as (−)-abietic acid, transformable into (+)-dehydroabietic acid, and (+)-dehydroabietylamine [23,24]. This makes this family of compounds very attractive for further drug development due to their broad pharmacological and drug-likeness properties. To the best of our knowledge, only one abietane-based commercial drug exists, ecabet sodium (**2**, Figure 1), available in Japan to treat acid peptic disorders [25].

The combination of drugs has been historically successful for severe and chronic viral diseases such as those originating from human immunodeficiency virus (HIV) and hepatitis C virus (HCV) infections and is a developing strategy for emerging or re-emerging viral diseases, including severe acute respiratory syndrome coronavirus (SARS-CoV), Middle East respiratory syndrome (MERS)-CoV, Zika, Ebola, and influenza [26]. These combinations include abacavir/dolutegravir/lamivudine (triumeq), darunavir/cobicistat/emtricitabine/tenofovir (symtuza), ledipasvir/sofosbuvir, sofosbuvir/velpatasvir, and lopinavir/ritonavir (kaletra). Many other drug combinations are currently in clinical trials against SARS-CoV-2, HCV, HBV, HSV-1, and other viral infections [27]. Studies of drug–drug interactions through in vitro drug combination assays are important for several reasons. High doses of certain drugs can cause adverse effects, whereas combining drugs may facilitate the use of lower doses while maintaining the desired therapeutic outcome or even enhancing it due to synergistic effects. Additionally, combining drugs with different mechanisms of action can help prevent the development of resistance by attacking the infectious agent through multiple pathways [26]. Concurrent agents that target virus entry and virus replication, called directly acting antivirals, and cellular proteins involved in and necessary for viral replication, called host-targeting antivirals, offer opportunities to discover synergistic drug combinations [22,28,29].

CHIKV and ZIKV are RNA genome re-emerging viruses that are part of the group of arboviruses, which are transmitted by arthropod vectors. These viruses have created a new challenge for public health in the Americas, with CHIKV emerging in late 2013 and ZIKV in 2014, and their infections have spread globally, causing a spectrum of diseases that ranges from self-limited febrile illness to permanent severe disability, congenital anomalies, and early death [30]. Diseases caused by arboviruses such as CHIKV, ZIKV, and dengue virus (DENV) can produce very similar clinical symptoms, mainly during the acute phase (the first days of the disease), hindering clinical diagnosis by health workers, creating problems for appropriate and early case management, and sometimes triggering fatal events [30]. According to the World Health Organization, almost 4 billion people live in areas where arboviruses are current public health threats. HHV stands out for being the main virus responsible for many infections in the orofacial region as well as in the genital region. HHV is the prototype of a large family of double-stranded DNA genome viruses; human herpesvirus (HHV) types 1 and 2 establish latent infections in sensory neurons and undergo reactivation during periods of severe host immunosuppression. Prolonged therapy with acyclovir (ACV) and its analogs can lead to drug-resistant HHV strains in patients infected with the human immunodeficiency virus (HIV) [31]. Continued research in virology and pharmacology will predictably lead to the identification of new molecular targets for clinical intervention, with the objective of developing new combined treatments that will produce therapeutic synergies in the future [31,32]. The importance of developing an integrated approach and networking in the study of natural products for advancing new therapies is essential, and The International Natural Product Sciences Taskforce’s (INPST) use of digital communications tools is a clear example of this [33].

Given the known antiviral properties of IVM [3,4,5,6], some EOs [8], and phthFGL [18,19,20,21] in certain viruses, we envisioned exploring the resultant antiviral activity of different combinations of commercially available IVM, commercially available oregano, or fennel essential oils, and phthFGL synthesized in our laboratory. The aim of this study was to evaluate a possible synergistic effect among the different substance combinations for the in vitro inhibition of the replication of Chikungunya virus (CHIKV), herpesvirus (HHV), and Zika virus (ZIKV), as well as reductions in adverse effects in our cellular model (Vero-E6 cells) due to IVM determining cytotoxicity in vitro.

## 2. Results

### 2.1. Antiviral Evaluation of IVM, Eos, and phthFGL 1a against ZIKV, CHIKV, and HHV-2

To evaluate the potential antiviral synergism in vitro that may arise from drug combination, it is necessary initially to determine the effective concentration that inhibits 100% of the infection in cell cultures. Additionally, to calculate a selectivity index (SI), it must be correlated with the cytotoxic concentration, i.e., the one that causes a 100% loss of cell viability. To determine the cytotoxic concentration, the crystal violet technique was used. Briefly, 2.0 × 10^3^ cells/well of Vero-E6 cells were grown in 96-well flat-bottomed plates at 37 °C in a humidified 5% CO_2_ atmosphere. After 24 h of incubation, two-fold dilutions of the compounds and the respective positive controls for each evaluated virus were added. The most pertinent concentration units were used for both positive controls and molecules under study, depending on their starting state. After 48 h of incubation, the cell monolayers were stained, and the concentration that completely detached the cell monolayer, defined as the concentration cytotoxic 100 (CC_100_), was visually identified (Table 1).

The same protocol was used to evaluate the antiviral concentration that inhibits 100% of the cytopathic effect, with an additional step of infecting the cell monolayer with 10 median tissue culture infection dose (10 TCID50) of the respective virus before adding non-cytotoxic two-fold dilutions of the compounds (these two steps were performed almost simultaneously). The minimum inhibitory concentration that did not allow visualizing plaque formation as a cytopathic effect caused by the virus was called the 99% inhibitory concentration (IC_99_) (Table 1). Using IC_99_ has many advantages over the IC_50_; IC_99_ reduces the number of experiments, eliminates the need to ensure a dose-dependent effect, and is a fast and inexpensive method that does not require additional statistical analysis. Previously, the antiviral activity of each compound had to be known, ensuring 100% inhibition of the cytopathic effect through visual determination (and dose-dependent effect). The methodology standardized here is only an approximation, equivalent to a “synergism screening” where synergism controls should be included in the experimental design. As a positive synergism control, we included the combination of RIBA with ACV in the viral model of HHV-2, a synergism that has been documented by several studies [34,35]. As can be seen in Table 1, the IC_99_ of RIBA, in the presence of ACV (at the concentration of 1.3 µM), was reduced from 176 to 36.6 µM, a reduction that is equivalent to 4.8-fold its initial concentration. This parameter of higher than “four-fold” was chosen as a significant threshold when analyzing potential synergism in the binary or ternary combinations tested in this study. As shown in Table 1, the tested compounds IVM, **1a,** and EOs have lower SI values than the selected positive controls ACV and heparin. IVM had the lowest selectivity index (of 2 against HHV-2), while phthFGL (**1a**) showed the highest IC_99_ value (SI = 16). Broad-spectrum antiviral activity has already been demonstrated for EOs, but this is the first time that their antiviral activity, specifically that of oregano and fennel Eos, has been reported for both HHV-2 and arboviruses. Likewise, it is the first time that IVM has shown activity against HHV-2 replication; but the IC_99_ value could not be determined, since it was close to the CC_100_ value. In our experimental design, binary or three-component combinations were tested to demonstrate whether the 100% inhibitory effect is maintained with a reduction in the concentration of IC_99_. The increase in SI was also analyzed.

### 2.2. Antiviral Evaluation of Binary and Ternary Combinations of Compounds

**Figure 2:**
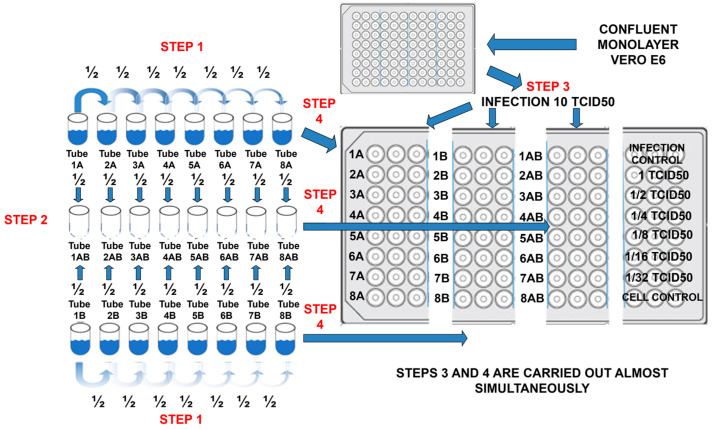
Experimental workflow design for synergism determination.

**Figure 3:**
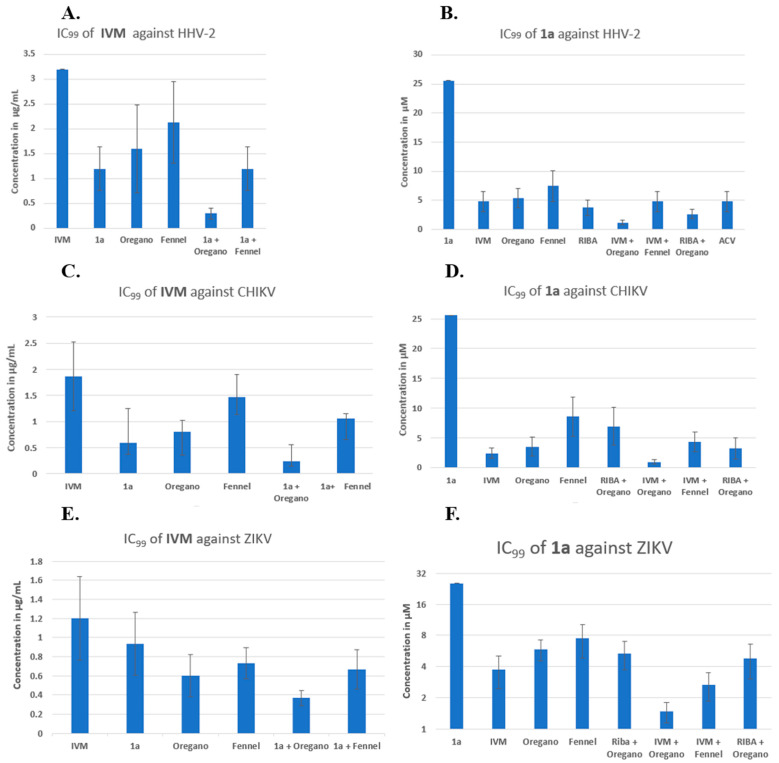
Reduction in IC_99_ inhibitory concentration of IVM or compound **1a** against HHV-2 virus (**A**,**B**), CHIKV (**C**,**D**), and ZIKV (**E**,**F**) in binary and ternary combinations, respectively.

In Figure 2, the general experimental workflow design for determinations of synergisms is shown. Briefly, in a 96-well flat-bottomed plate were transferred (step 4) two-fold dilutions (step 1 and 2) of the compounds and mixed compounds to cultured confluent monolayer Vero-E6 cells which have been infected (step 3) with viral suspension (10TCID50/0.1 mL). Viral titer control is found in the upper right panel, which consisted of two-fold dilutions, starting at 1TCID50, and finalizing in the 1/32 TCID50 dilution. The lower right panel was the cell control. The center-right panel contained the double serial combination of two compounds. The IC_99_ data were recorded with three replicates for each compound (the IC_99_ average concentrations are already reported in Table 1) and the mixture of compounds. Three-component combinations reduced IC_99_ concentration more than binary combinations. Two experiments by triplicate for each viral type and each concentration (binary and three-component combinations) were tabulated and graphed with their standard deviation, as shown in Figure 3.

#### 2.2.1. Antiviral Activity of IVM or phthFGL (1a) in the Context of Combinations against HHV-2, CHIKV, and ZIKV

In Figure 3A (see overleaf), the IVM concentration in µg/mL is plotted on the *y*-axis, and the order of presentation on the *x*-axis is as follows: IVM, IVM/**1a**, IVM/oregano, IVM/fennel, IVM/(**1a** + oregano), and IVM/(**1a** + fennel). Figure 3A shows that binary combinations reduced the IVM inhibitory concentration, as the shown combination IVM/fennel displayed the least positive effect. In the last two combinations, the IVM/(**1a** + fennel) combination resulted in approximately the same effect as the binary combination IVM/**1a**. The most potent effect observed was from the ternary combination, IVM/(**1a** + oregano), which was greater than that of any binary combination. It should be noted that IVM alone was not able to inhibit 100% of lysis plaque formation by HHV-2 infection (the bar represents approximately 50%); this was only possible by combining with other compounds. It is clear that binary and ternary combinations of agents have potential, but the resultant data indicate that the reduction in IC_99_ is made significantly greater by the additional contribution of oregano EO in combination with two other components. In Figure 3B, the compound **1a** concentration in µM is plotted on the *y*-axis, and the order of presentation on the *x*-axis is as follows: **1a**, **1a**/IVM, **1a**/oregano, **1a**/fennel, **1a**/RIBA, **1a**/ACV, **1a**/(IVM + oregano), and **1a**/(IVM + fennel). The binary combinations of **1a** with IVM, oregano EO, fennel EO, RIBA, and ACV reduced the IC_99_ of compound **1a** generally around five times from 25.6 to ca. 5 µM. The three-component **1a**/(IVM + oregano) and **1a**/(IVM + RIBA) mixtures reduced the concentration much more at values below 5 µM; the oregano-based ternary mixture including IVM makes a big difference in the reduction of IC_99_. For combinations of three compounds, the descending order of effectiveness was: **1a**/(IVM+ oregano) > **1a**/(oregano+ RIBA) > **1a**/(IVM+ fennel). The IC_99_ reduction by binary and/or three-component combinations was more evident when the analysis was performed from **1a** (Figure 3B). This could be because **1a** has the highest value of IC_99_ concentration, and the two-fold dilutions used show per se a greater effect (12.8, 6.4, 3.2, 1.6, 0.8, 0.4). Control drugs (RIBA and ACV) were combined with **1a**; both compounds reduced the IC_99_ of **1a** from 25 µM to approximately 5 µM. In the subsequent experiments for the CHIKV and Zika viral models, only RIBA was used as a control since this drug possesses broad-spectrum properties.

CHIKV (with an RNA genome) was another viral model for the study of binary and three-component combinations (Figure 3C,D), which was much more sensitive to the binary combination of IVM/**1a** than HHV-2 (Figure 3C/second column). IVM had an IC_99_ of 1.8 µg/mL (Table 1, Figure 3C/first column), and the reductions in this concentration in the binary combinations IVM/**1a** and IVM/oregano EO were below 1.0 µg/mL. The combination IVM/fennel EO was the least effective, as it was also the case with the three-component combination of the latter with **1a**. Also, the effect of the combination IVM/oregano EO + **1a** was greater than that of IVM/fennel + **1a**. As stated for Figure 3B, when making the respective analysis with **1a**, Figure 3D shows the same tendency for both the binary and three-component combinations. However, in this case, CHIKV is more sensitive to the binary combination **1a**/oregano EO than **1a**/fennel EO.

Another RNA genome model, ZIKV (Figure 3E,F), was also studied and shown to be less sensitive than CHIKV, but of all the binary combinations, the one containing oregano EO was the most effective in reducing the IC_99_ of IVM (Figure 3E). Also, the effect of the combination IVM/**1a** + oregano EO was greater than that of the other ternary mixture, IVM/**1a** + fennel. In Figure 3F, the respective analysis with compound **1a** is depicted. In this case, ZIKV was just as sensitive as CHIKV. However, the effect of the combination **1a**/ RIBA + oregano EO was less sensitive than that of **1a**/IVM + fennel. For the combinations of three agents, the following descending order of efficiency was observed: **1a**/(IVM+ oregano) > **1a**/(IVM+ fennel) > **1a**/oregano + RIBA.

#### 2.2.2. Reduction in IC_99_ Values in Comparison with ACV/Ribavirin (ACV/RIBA) as Synergism Control

**Figure 4:**
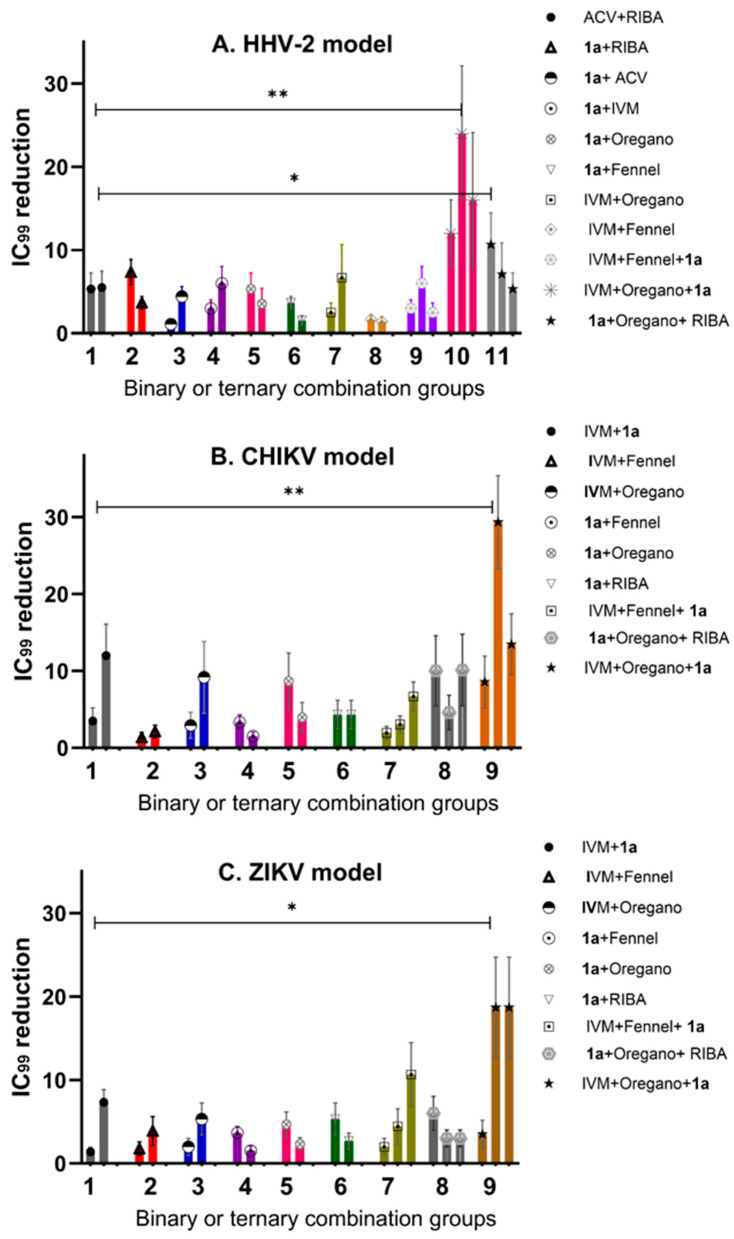
Reduction in IC_99_ inhibitory concentration (number of times) for the different models (**A**) (HHV-2), (**B**) (CHIKV), and (**C**). ZIKV in binary and ternary combinations. Differences in *p*-value greater than 0.05 were considered statistically not significant (n.s.), while groups with *p*-values of significance are indicated by ** for *p* = 0.0098 and * for *p* = 0.0390.

To have the same arbitrary unit of comparison to evaluate the potential synergisms, the *y*-axis is indicated by the fold change defined as a quantity change between the original and a subsequent measurement of the IC_99_ value. Figure 4 shows the calculated outcome of the studies comparing the three viral models, indicating the importance of combinational therapy. The ACV/RIBA system was selected as the synergism control or minimum threshold, which reduced the IC_99_ value four-fold in the HHV-2 model. Thus, any combination that reduces the IC_99_ value by more than four-fold would be considered as a mixture with a potential synergism effect. Figure 4A indicates the IC_99_ reduction values for antiviral activity against the HHV-2 virus. The experimental design clearly indicates which evaluated combinations have synergisms effects. The control: ACV + RIBA, ACV + **1a**, and RIBA + **1a** were near the 5-fold threshold line, except compound **1a** mixed with ACV (Figure 4A). The binary combinations: IVM + fennel and **1a** + fennel were also below the 5-fold threshold line. The combination of three components that showed the best IC_99_ value against HHV-2 was IVM + **1a** + oregano EO, which indicated reductions of more than ten, twenty, and fifteen times, respectively. The combination RIBA+ **1a** + oregano EO showed a slightly larger value than the binary combination RIBA + **1a**, but a much smaller one than that of IVM + **1a** + oregano, which suggests that the mechanism of action of IVM (inhibition of host heterodimeric IMP α/β1 complex) might be of greater importance for viral survival since it makes a higher contribution to certain cell targets than RIBA.

The RNA genome model CHIKV was the one that showed the greatest sensitivity at synergism, as can be seen in the combination of three components, where the **1a** IC_99_ value was reduced more than twenty-five times (Figure 4B). Unfortunately, all binary combinations were below the 5-fold threshold line; the only exceptions were those containing compound **1a** where the synergism produced a reduction in the IC_99_ value from five to ten times when it was mixed with oregano EO or IVM, respectively. In the same way as shown with the HHV-2 model, the differences between the two EOs are conserved in the CHIKV model, with oregano-EO-containing mixtures displaying the greatest interaction to promote synergism. On the contrary, the ZIKV model was the least sensitive for the reduction in the IC_99_ value, with almost all the binary combinations falling below the 5-fold threshold (Figure 4C), except those with compound **1a,** which reduced the IC_99_ value by more than five times when mixed with IVM. In the discussion section, the plausible mechanism of action that has been evaluated so far for compound **1a** will be analyzed, considering what is known about its cellular or viral targets with respect to targets of the other compounds tested. If we compare the two RNA genome models, there are differences in the results obtained by the combinations of three components, mainly when the difference in the mixture was the EO used. In addition, for viruses of the RNA genome, it can be concluded that oregano-EO-containing mixtures present a greater reduction in IC_99_ values, which suggests that the chemical composition of oregano EO possibly facilitates more synergism in the routes used by CHIKV and ZIKV for their replication than in those of HHV-2.

One-way ANOVA followed by Tukey’s post hoc test were conducted to compare the effect of IC_99_ reduction on different groups of combinations in the different models, including groups of two-component mixtures and ternary combinations. There was a significant difference in IC_99_ reduction (number of times) among the different combinations at the *p* < 0.05 level for all the groups, which was especially relevant in the case of the HHV-2 and CHIKV models with *p* = 0.0098 depicted as ** (Figure 4).

The composition of the EOs was analyzed and reported by the company “doTERRA” for the batch supplied (oregano ID 171778 and fennel ID 161894, company website www.sourcetoyou.com). Briefly, the major components of oregano EO versus fennel EO are 74.2% carvacrol vs. 74.5% trans-anethole. The minor components are, for oregano EO, linalool, thymol, beta-caryophyllene, terpinene-4-ol, and alpha- and gamma-terpinene; and for fennel EO, limonene, alpha-pinene, fenchone, methyl-chavicol, and beta-caryophyllene.

## 3. Discussion

Due to the high costs associated with drug development, there is an imperative to search for new strategies. Drug combination therapies are one of these new strategies to combat emerging viral infections, among other types of diseases. By using faster and more economical screening methods, candidates with “effective synergies” could be rapidly identified, facilitating or enabling faster paths to clinical testing [26,27,28,29]. IVM is a specific inhibitor of the importin-α/β-dependent nuclear transport protein complex (IMPα/β1) and has shown potential antiviral activity against several RNA and DNA viruses [2,3,4,5,6]. For example, IVM’s antiviral action on coronavirus is explained as follows: during SARS-CoV-2 replication, the IMPα/β1 complex binds to coronavirus cargo proteins in the cytoplasm translocating them through the nuclear pore complex (NPC) to the nucleus [2,6]. There, the viral proteins reduce the antiviral response of the host cell, leading to enhanced infection [2,3,4,5,6]. IVM dissociates preformed IMPα/β heterodimers, which are required for the nuclear import of coronavirus proteins. This prevents IMPα/β1 from binding to viral proteins and entering inside the nucleus [6,36]. Caly et al. (2020) [2] tested IVM’s antiviral activity against SARS-CoV-2 (Australia/VIC01/2020 isolate) in Vero/hSLAM cells at a multiplicity of infection (MOI) of 0.1 (48 hpi) resulting in an EC_50_ value of ca. 2.0 μM [2]. High doses of 600 μg/kg/day of IVM in humans have been shown to produce a maximum plasma concentration (Cmax) of 120 ng/mL (~0.14 μM) [37], which is much lower than its in vitro antiviral value of ~2 μM [2]. This could be supported by simulation studies obtained in mathematical models which have estimated that IVM achieves lung tissue concentrations up to three times higher than plasma concentrations [37]. However, a 5-day treatment with 12 mg once daily of IVM in adults showed viral clearance in SARS-CoV-2 patients at 9.7 days compared to 12.7 days in the placebo group [38]. Moreover, in a clinical study with dengue patients (DENV4/45.2%, DENV3/33.9%, DENV2/8.7%, and DENV1/7.0%), a daily oral dose of 400 µg/kg of IVM also showed a trend towards a reduction in plasma nonstructural protein 1 (NS1) clearance at day 3 compared to placebo, though clinical efficacy was not observed with that dosing regimen [39], even though in vitro, IVM EC_50_ values for DENV/1-4 ranged from 1.6 to 2.3 μM in BHK-21 cells (48 hpi) [40]. To date, there remains a discrepancy regarding the applicability of IVM as a broad-spectrum antiviral treatment due to its low plasma levels achieved with oral treatments. For this reason, drug combination synergism may be a plausible alternative [41,42], as well as innovations in administration routes (such as nasal spray formulations [43]) to enhance its effectiveness.

In this study, we standardized a rapid method to identify possible synergisms from drug combinations analyzing the possible reduction in effective antiviral concentration of IVM and other candidate molecules already approved by the FDA. EOs were included in the study because they have been reported for more than a couple of decades to have broad-spectrum antiviral activity [8,11,12,13,14,15]. In the experiments, we added one of our patented molecules with demonstrated broad-spectrum in vitro antiviral activity [20], the abietane-derived analog 18-(phthalimide-2-yl)ferruginol (phthFGL) (**1a**, Figure 1), as a continuation of our studies on this “wonder” molecule. In light of the present research, evidence of broad-spectrum antiviral activity (against ZIKV, CHIKV, and HHV-2) of different combinations of three molecules (IVM, RIBA, and phthFGL) and two EOs (oregano and fennel) is reported. IVM had already shown activity against ZIKV and CHIKV [6,44]. We found an IC_99_ value of ca. 1.2 μg/mL (1.37 μM) for ZIKV in a period of 48 hpi (10 TCID50/~MOI= 1). Also, Varghese et al. (2015) [44] reported an anti-CHIKV IC_50_ value of 0.6 μM in BHK-21 cells at 16 hpi/MOI = 0.01, while the CHIKV IC_99_ value obtained by us was 1.8 μg/mL (2.1 μM). To the best of our knowledge, IVM has not been studied against HHV-2 until now. In this study, IVM was less active against HHV-2 than against ZIKV and CHIKV. Słońska et al. (2013) [45] evaluated the antiviral activity of IVM against two strains of equine herpesvirus type 1 (Jan-E EHV-1 and Rac-H EHV-1) but only antiviral activity against the Jan-E EHV-1 strain was found. Lv et al. (2018) also investigated the effect of IVM on virus replication of a subfamily alpha-Herpesviridae such as pseudorabies virus (PRV) [46]. They found that IVM at 2.5 μM disrupted the nuclear localization of PRV UL42 (accessory subunit of PRV DNA polymerase) in a BHK-21 cell line model, by targeting the nuclear localization signal of the proteins, and decreased PRV titers by more than 7000-fold after 48 h of viral infection [46]. Döhner et al. (2018) suggest that importin α1 protein is specifically required for the nuclear localization of several important HHV-1 proteins involved in processes such as capsid assembly and capsid egress into the cytoplasm [47]. In our study, we found that IVM had antiviral activity against HHV-2 at concentrations below 3.2 μM, reaching approximately up to 50% inhibition. It was not possible to find an IC_99_ concentration because its value was close to the cytotoxic concentration in our experimental model (commercial-IVM/Vero E6 cells). Our results of the antiviral activity of IVM against HHV-2 allow us to assume that a subfamily of alpha-Herpesviridae, the HHV-2, use nuclear carriers for their viral proteins as it has been shown by other researchers for related viruses [45,46,47].

PhthFGL (**1a**, Figure 1) is an abietane-derived analog of (+)-ferruginol (**1**, Figure 1), structurally similar to the commercial drug ecabet sodium (**2**, Figure 1) [20,48]. To date, ecabet sodium is the only commercial drug based on abietane-type diterpenoids which is clinically used in the treatment of gastritis and gastric ulcers in Japan since it possesses high affinity to gastric adherent mucosa, epithelial cells, albumin, and fibrinogen in the ulcer region [48]. Following a single oral administration of an ecabet disodium tablet (1 g) volunteers showed a plasma maximum concentration–time profile of 4926 ± 880 ng/mL at the first hour and a plasma elimination half-life of 6.63 ± 2.24 h [48,49]. Meanwhile, for ferruginol (**1**, Figure 1), pharmacokinetic studies have been performed in rats obtaining a maximum concentration in plasma of 3140 ng/mL at 40 min after oral administration at a dose of 20 mg/kg (~1 g in adults), and a plasma elimination half-life of 41.73 min [50]. In silico modeling studies of drug-likeness, such as Lipinski’s rules, have reported that both ferruginol and phthFGL violate only one rule, which corresponds to the partition coefficient (its values are 6.41 and 7.08, respectively, and it cannot be greater than 5) [21]. In addition, in silico modeling prediction of toxicity, using the Gosselin, Smith, and Hodge scale, indicated that phthFGL possesses a reasonably good safety profile when administered orally in rats [21]. The predicted LD_50_ value for phthFGL expressed as the weight of the chemical per unit of body weight was in the range of 500 < LD_50_ ≤ 5000 mg/kg, thus being moderately toxic. According to the Organization for Economic Co-operation and Development (OECD) manual, it was classified as class 4 [21]. During different research studies, we have reported the antiviral activity of phthFGL against DENV-2 and HHV-2 [18], ZIKV [19,20], and CHIKV [20]. This interesting molecule displayed EC_50_ values in the range of 1.0–20.0 µM for those viruses, specifically, 1.4 µM for DENV-2 and 19.2 µM for HHV-2 [18], 7.7 µM for a Brazilian Zika strain (clinical isolate, IMT17) [19], 5.3 µM for COL345Si Zika, 6.3 µM for Zika_459148, and 9.8 µM for CHIKV [20]. Very recently, we demonstrated that it was also active against human coronavirus HCoV 229E (PHE/NCPV 0310051v) where an IC_99_ value of 3.0 µg/mL (6.9 µM) was found [21]. In the experimental design of this study, double serial dilutions were used from 0.4 to 25.6 µM for phthFGL, resulting in IC_99_ values of 25.6 µM for the three tested viruses ZIKV, CHIKV, and HHV-2. Unpublished studies strongly suggest that implicated in the antiviral mechanism of action of phthFGL is the disruption of the viral polyprotein translation, via alteration in actin remodeling, as well as other related cellular and viral processes involved in the replicative complex formation [18,22].

DeForni et al. (2022) [51] evaluated the effect of combining IVM, remdesivir (RDV), and azithromycin (AZI) on SARS-CoV-2 replication in vitro. In that study, a two-dimensional matrix of 49 different combinations to quantify synergistic interactions was created to validate synergistic concentrations. They mixed two-fold serial dilutions of compounds with Vero E6 cells (~70% confluence) in 96-well assay plates; 1 h later, the cells were infected with SARS-CoV-2 at a multiplicity of infection MOI = 0.01. These researchers found that combining RDV and IVM led to lower concentrations required to achieve complete inhibition (~IC_99_), specifically 6- and 13-fold for RDV and IVM, respectively [51].

In a similar manner, we also used two-fold serial dilutions of compounds (IVM and phthFGL, or oregano or fennel EOs) and mixed them with the 10 TCID50 of each virus (CHIKV, ZIKV, and HHV-2). Then, these mixtures were added to 96-well assay plates when Vero E6 cells reached ~80% confluence. The IVM and phthFGL combination led to much lower IC_99_s than either single treatment or IVM combined with oregano or fennel EOs. The combined IC_99_ of IVM and phthFGL against HHV-2 was 1.2 ± 0.4 vs. >3.5 µg/mL with only IVM (Figure 3A), while for CHIKV the IC_99_ was 0.6 ± 0.2 µg/mL vs. 1.8 ± 0.6 µg/mL (Figure 3C), and for ZIKV the IC_99_ was 0.93 ± 0.3 vs. 1.2 ± 0.4 µg/mL (Figure 3E), in this drug combination regimen. The synergism controls such as RIBA and ACV reported by Pancheva and co-workers were used to validate the results obtained with our own experimental design [34,35]. RIBA and ACV were evaluated against HHV-2 as a positive control for the model of the “potentiating effect” of synergism since the two drugs exhibit complementary mechanisms of action [34,35]. On the one hand, it is known that ACV is selectively phosphorylated by herpes simplex virus (HHV)-encoded thymidine kinase, and then, cellular enzymes catalyze the conversion of ACV-monophosphate to its triphosphate form (ACV-TP) [52]. ACV-TP inhibits viral DNA synthesis and leads to inactivation of the HSV-l-encoded DNA polymerase [52]. On the other hand, RIBA, among other mechanisms, inhibits the synthesis of guanosine monophosphate, consequently reducing the intracellular guanine nucleotide pool (GTP and dGTP, the natural counterpart of ACV-TP, [52]), an action that may explain its antiviral activity against both DNA and RNA viruses [34,35,52]. In the HHV-2 viral model, the IC_99_ for RIBA was reduced from 176 µM to 36.6 ± 10 µM when it was combined with ACV, while for ACV, the IC_99_ decreased from 6.6 µM to 1.3 ± 0.4 µM, which is the ca. 5-fold threshold in the synergism determinations (Figure 4A). Pancheva et al. (1991, 1990) found an IC_50_ reduction to 40 µM by RIBA combined with ACV (1.1 µM) against HHV-1 [34,35], results that agree with what we obtained in our experimental design for these two controls. Moreover, RIBA combined with phthFGL (**1a**) reduced its IC_99_ value from 176 µM to 51.3 ± 39 µM while for ACV the IC_99_ value decreased from 6.6 µM to 1.87 ± 0.6 µM. Therefore, the most favorable binary combination to reduce the IC_99_ value was the one presented with **1a**, even when it was evaluated together with RIBA (25.6 µM vs. 3.73 ± 1.19 µM). Experimental studies carried out by us [11] and other researchers [10,12,13,53] indicate that EOs interfere with virion envelope structures masking viral envelope proteins, which are necessary for adsorption and entry into the host cell. Mediouni et al. (2020) [53] found that the anti-HIV-1 activity of oregano EO depends both on the composition, logically, and on the virus envelope. The anti-HIV-1 activities of the main oregano EO components, carvacrol and its isomer thymol and their mixtures, were explained as a virucidal activity, where these components inactivated the virus binding site (glycoprotein binding peptide) of viral gp120 to its host cell [53]. Carvacrol also changes the proportion of cholesterol present in the viral envelope (or cell membranes) [10,53]. It has been shown that the virus modulates cholesterol metabolism during the viral cycle, affecting intracellular cholesterol homeostasis [10,53,54,55]. From a previous study of the antiviral activity of more than thirty EOs from species of the Verbenaceae, Piperaceae, Poaceae, Lamiaceae, Lauraceae, and Myrtaceae families against ZIKV, DENV, CHIKV, HHV-2, and HHV-1 viruses, of which only the results against CHIKV have been published [56], the oregano and fennel EOs were selected for the present study. The *Origanum vulgare* (oregano) and *Foeniculum vulgare* (fennel) EOs from the doTERRA company showed virucidal activity with broad-spectrum antiviral properties. Based on this background, it was decided to include EOs in our synergism studies.

In this study, the combination of IVM, phthFGL, and oregano EO showed the greatest synergism potential with higher values for CHIKV than ZIKV and HHV-2, obtaining a reduction in the EC_99_ value of up to ~8 and ~27-fold for IVM and phthFGL, respectively, while for oregano EO the reduction was ~12-fold. The synergism potential exhibited by oregano EO can be explained by its high carvacrol content. As it was explained above, carvacrol displaces cholesterol molecules in the cell membrane [53]. EOs act directly on the respiratory, circulatory, and central nervous systems through the skin and respiratory tract, and the administration in spray form has recently been considered as a new strategy for EO therapeutic application [57]. Recently, nasal IVM spray administration in a pig model has been shown to attain high drug concentrations in nasopharyngeal tissue, a primary site of aerosol-spread virus entrance/replication [43]. In this study, we demonstrated the synergy among the components IVM, phthFGL, and oregano EO which act through different mechanisms of action against CHIKV, ZIKV, and HHV-2 in vitro.

A comparison of pharmacokinetics parameters simulated with the webserver SWISSADME for phthFGL (**1a**, Figure 1) and the commercial drug ecabet sodium (**2**, Figure 1) features similar and good drug-likeness characteristics (see Supplementary Materials, Figures S1 and S2). This supports phthFGL as a good lead candidate for the development of new broad-spectrum antivirals, which encourages further studies to improve the compound’s physicochemical properties (solubility, pH = 7.4 < 0.1 μg/mL). In fact, we checked the good pharmacokinetic profile of phthFGL through in vitro ADME studies (Tables S1 and S2, Supplementary Materials) following standard methods as reported previously by us and using various known drugs as controls [58].

## 4. Materials and Methods

### 4.1. Biological Assays

#### 4.1.1. Reagents and Compounds

Dulbecco’s modified Eagle’s medium (DMEM), L-glutamine, non-essential amino acids and minimum essential medium vitamin solution, NaHCO_3_, carboxymethylcellulose sodium salt medium viscosity (CMC), and 3-(4,5- dimethylthiazol-2-yl)-2,5-diphenyl tetrazolium bromide (MTT) were obtained from Sigma-Aldrich Chemical Co. (St. Louis, MO, USA). Fetal bovine serum (FBS) and penicillin/streptomycin were purchased from Invitrogen Life Technologies (Carlsbad, CA, USA). Ribavirin and acyclovir were obtained from Calbiochem (La Jolla, CA, USA). 18-(Phthalimide-2-yl)ferruginol (**1a**, phthFGL) was re-synthesized following the method for its practical synthesis conveniently described by us [20]. PhthFGL, acyclovir, and ribavirin stock solutions were prepared in dimethyl sulfoxide (DMSO, Sigma, Cream Ridge, NJ, USA) to be evaluated immediately. The essential oils (EOs) extracted from commercial aromatic plants were donated by Carmen Orozco, a distributor in Colombia of the company doTERRA-Essential Oils (Pleasant Grove, UT, USA). The GC-MS chromatographic analysis information was provided and the following EOs were requested: *Origanum vulgare* (oregano) and *Foeniculum vulgare* (fennel). The EOs have the ID quality codes oregano ID 171778 and fennel ID 161894, allowing us to search for their composition on the company’s website (www.sourcetoyou.com). Ivermectin 0.6% MK^®^ (MK Drug Vademecum- 6 mg/mL in an oral solution containing excipients q.s.) was obtained from the laboratory FARMATODO (Bogotá, Colombia). Both EOs and IVM were prepared in DMEM to be evaluated immediately. Before use, all reagents were kept refrigerated at temperatures no higher than 2 °C.

#### 4.1.2. Cell Culture and Viruses

Vero-E6 cells (African green monkey kidney-Cercopithecus aethiops, ATCC CRL-1586) were maintained in DMEM supplemented with 5% of inactivated fetal bovine serum (FBS), 100 units/mL of penicillin, 100 mg/mL of streptomycin, 100 mg/mL of L-glutamine, 0.14% NaHCO_3_, and 1% of each non-essential amino acids and minimum essential medium vitamin solution (choline chloride, D-calcium pantothenate, folic acid, nicotinamide, pyridoxal hydrochloride, riboflavin, thiamine hydrochloride, and i-inositol). The cells were incubated at 37 °C in a humidified 5% CO_2_ atmosphere for their maintenance. Zika virus_459148 (clinical isolate, Zika_virus_459148_Meta_Colombia_2016/GenBank-MH544701.2) and CHIKV were donated by the Virology Group “Dirección de Redes en Salud Pública” (Instituto Nacional de Salud, Bogotá, DC, Colombia). Zika virus_459148 characterization was described by Laiton-Donato et al. (2019) [59]. Human Alphaherpesvirus type 2, HHV-2 (VR-734), was purchased from the Center for Disease Control (Atlanta, GA, USA). Virus stocks were produced and titrated in Vero-E6 cells by plaque assay and expressed as plaque forming units (PFU/mL), or TCID50/0.1 mL, which means the dilution of the virus required to obtain 50% lytic effect of the cellular culture in 100 µL of viral suspension. TCID50/0.1 mL titrations were evaluated at 48 h post-infections in 96-well flat-bottomed plates, and the viral stops were frozen in liquid nitrogen at concentrations of 1 × 10^4^ TCID50/0.1 mL in a culture medium without serum (FBS).

#### 4.1.3. End-Point Titration Technique (EPTT) for Evaluation of IC_99_

The EPTT technique (Vlietinck et al., 1995) [60] was used with few modifications. The unit used in the EPTT assay for the three viruses (CHIKV, ZIKV, and HHV-2) was 10TCID50, which means the dilution of the virus required to obtain 100% lytic effect of the cellular culture in a well in 100 µL of viral suspension within 48 h of infection. For the evaluation of antiviral activity, initially, confluent monolayer Vero-E6 cells were grown in 96-well flat-bottomed plates (2.0 × 10^3^ cells/well), at 37 °C in a humidified 5% CO_2_ atmosphere. After 24 h of incubation (obtaining more or less 80% of the cell monolayer formed), the culture medium was removed. Then, viral suspensions (10 TCID50/0.1 mL) followed immediately by two-fold dilutions of the compounds were added in a maintenance medium, identical to the growth medium except for the FBS (at 1.0%). After 48 h of incubation at 37 °C in a humidified 5% CO_2_ atmosphere, the cell monolayers were stained with a solution of 3.5% formaldehyde with 0.2% crystal violet, and the cytopathic effect (CPE) was observed under an inverted microscope. The minimum inhibitory concentration that does not allow for visual detection of plaque formation as a cytopathic effect caused by the virus is called the 99% inhibitory concentration (IC_99_). Two independent experiments triplicated for each viral type and each concentration were carried out. Controls included untreated cells, cells treated with compounds, and cells infected with each viral type. The concentration of DMSO in assays was 0.05% and cellular controls with DMSO at 0.05% were used. The positive controls included were acyclovir (ACV), ribavirin (RIBA), and heparin (H). The values were expressed as the mean standard deviation (MSD).

### 4.2. In Silico Simulations

#### Calculation of Molecular Properties (Drug-Likeness)

The structures of compound **1a** and ecabet sodium **2** were manually drawn in ChemDraw Professional 22.2.0 software (PerkinElmer Informatics, Inc., Waltham, MA, USA), and the SMILES notation was obtained for each molecule. Then, the SMILES codes were introduced in the webserver SWISSADME [61] from the Swiss Institute of Bioinformatics (www.swissadme.ch) and calculation of physicochemical descriptors and druglike nature was performed.

### 4.3. Statistical Analysis

The statistical evaluation of the data for Figure 4 was carried out using the GraphPad Prism 10.00 software (Northside Dr, San Diego, CA, USA). All the samples were analyzed three times independently and the values reported were obtained as the mean of at least two independent experiments ± standard deviations (SD). Statistical evaluations were performed using one-way ANOVA followed by Tukey’s post hoc test.

Differences in *p*-value greater than 0.05 were considered statistically non-significant (n.s.), while groups with *p*-values of significance are indicated by ** for *p* = 0.0098 and * for *p* = 0.0390.

## 5. Conclusions

Arboviruses and respiratory viruses can produce very similar clinical symptoms in the first days of the disease, hindering clinical diagnosis by health workers, creating problems for appropriate and early case management, and sometimes triggering fatal events. The use of EOs as antivirals has several limitations, such as lack of knowledge on the exact mechanism of action, high volatility, certain instability which difficult drug formulations, and high lipophilicity leading to low solubility in biological fluids and consequently low bioavailability [8]. The main advantage of drug combinations is that if there are synergistic effects, the dosage is lower and thus there are lesser side effects, though the latter might be increased by drug–drug interactions. Another advantage is that if components of the drug combination have different mechanisms of action, then resistance mechanisms are reduced. Studies of drug combinations with broad-spectrum antiviral activity, as described in this report, provide a step towards more effective drugs against diseases caused by emerging viruses. In the future, further studies of drug–drug interactions with commercially available medications will enable individuals to quickly take some medications at home for a few days to prevent severe illness before being attended to by the governmental healthcare system. In this work, we demonstrated that a ternary combination of IVM, oregano EO, and a ferruginol analog, namely phthFGL (**1a**), is endowed with very promising antiviral properties, reducing several times the dosage of independent components needed to effect similar antiviral effects. The statistical analysis revealed very significant results, especially for the herpes and CHIKV models. However, achieving normalized practical application may need additional studies in comparison to normal single-drug development. Continued research in this area is crucial for improving our ability to respond to emerging virus threats [62].

## Figures and Tables

**Table 1:** Antiviral and cytotoxic activity of the compounds for the drug combination study.

		ZIKV		CHIKV		HHV-2	
Compounds	CC_100_	IC_99_	SI	IC_99_	SI	IC_99_	SI
1a, µM	410	25.6	16	25.6	16	25.6	16
IVM, µg/mL	6.4	1.2 ± 0.4	5.3	1.8 ± 0.6	3.5	3.2 *	2
Oregano, ppm	18.6 ± 0.6	8	2.3	3.6 ± 0.7	5.1	5.3 ± 1.8	3.5
Fennel, ppm	64	12 ± 4	5.3	14.6 ± 2.9	4.4	12 ± 4	5.3
RIBA, µM	>700	176	>4	44	>16	176	>4
ACV, µM	>660	NT	--	NT	--	6.6	>100
Heparin, U.I/mL	640	NT	--	NT	--	10	64
RIBA/ACV 1.3 µM	NT	NT	--	NT	--	36.6 ± 10	--

CC_100_: 100% cytotoxic concentration; SI: selectivity index; NT: not tested; --: not calculated; *: IVM was able to inhibit the cytopathic effect to approximately 50%, but at a concentration that showed a reduced cell monolayer.

## Data Availability

Data are contained within the article and raw data are available upon request.

## Supplementary Materials

The following supporting information can be downloaded at: https://www.mdpi.com/article/10.3390/ph16111602/s1, Figure S1: SWISSADME analysis for compound **1a**; Figure S2: SWISSADME analysis for compound **2**; Table S1: In vitro pharmacokinetic parameters for compound **1a**: Caco2 permeability; Table S2: In vitro pharmacokinetic parameters for compound **1a**: stability in microsomes and plasma.

## Sample Availability

A sample of compound **1a** is available from the authors.
