# Supplementary material for: Synergistic In Vitro Antiviral Effect of Combinations of Ivermectin, Essential Oils, and 18-(Phthalimid-2-yl)ferruginol against Arboviruses and Herpesvirus
Source: Pharmaceuticals (Basel). 2023 Nov 13;16(11):1602. doi: 10.3390/ph16111602 (PMC10674234; doi:10.3390/ph16111602)
Supplement: Supplementary file 1 [file pharmaceuticals-16-01602-s001.zip › pharmaceuticals-2683605-supplementary.pdf]

pharmaceuticals

Supplementary Information

Synergistic in vitro Antiviral Effect of Combinations of Ivermectin, Essential Oils and 18-(Phthalimid-2-yl)ferruginol Against Arboviruses and Herpesvirus

Liliana Betancur-Galvis [1,*], Orlando José Jimenez-Jarava [1], Fatima Rivas [2], William E. Mendoza-Hernández [3], and Miguel A. González-Cardenete [3,*]

[1] Grupo GRID- Grupo de Investigaciones Dermatológicas, Instituto de Investigaciones Médicas, Facultad de Medicina, Universidad de Antioquia, 050010 Medellín, Colombia

[2] Department of Chemistry, Lousiana State University, 133 Chopping Hall, Baton Rouge, Louisiana 70803, United States.

[3] Instituto de Tecnología Química, Universitat Politècnica de València-Consejo Superior de Investigacio-nes Científicas, Avda. de los Naranjos s/n, 46022 Valencia, Spain.

Email: migoncar@itq.upv.es

Contents

- Swissadme analysis for compound **1a** …………………………………………....Figure S1, page S2

- Swissadme analysis for compound **2** ………………….…………………….….…Figure S2, page S3

-Table S1: In vitro pharmacokinetic data **1a**: Caco2 permeability …........................Table S1, page S4

-Table S2: In vitro pharmacokinetic data **1a**: stability in microsomes and plasma…...Table S2, page S4

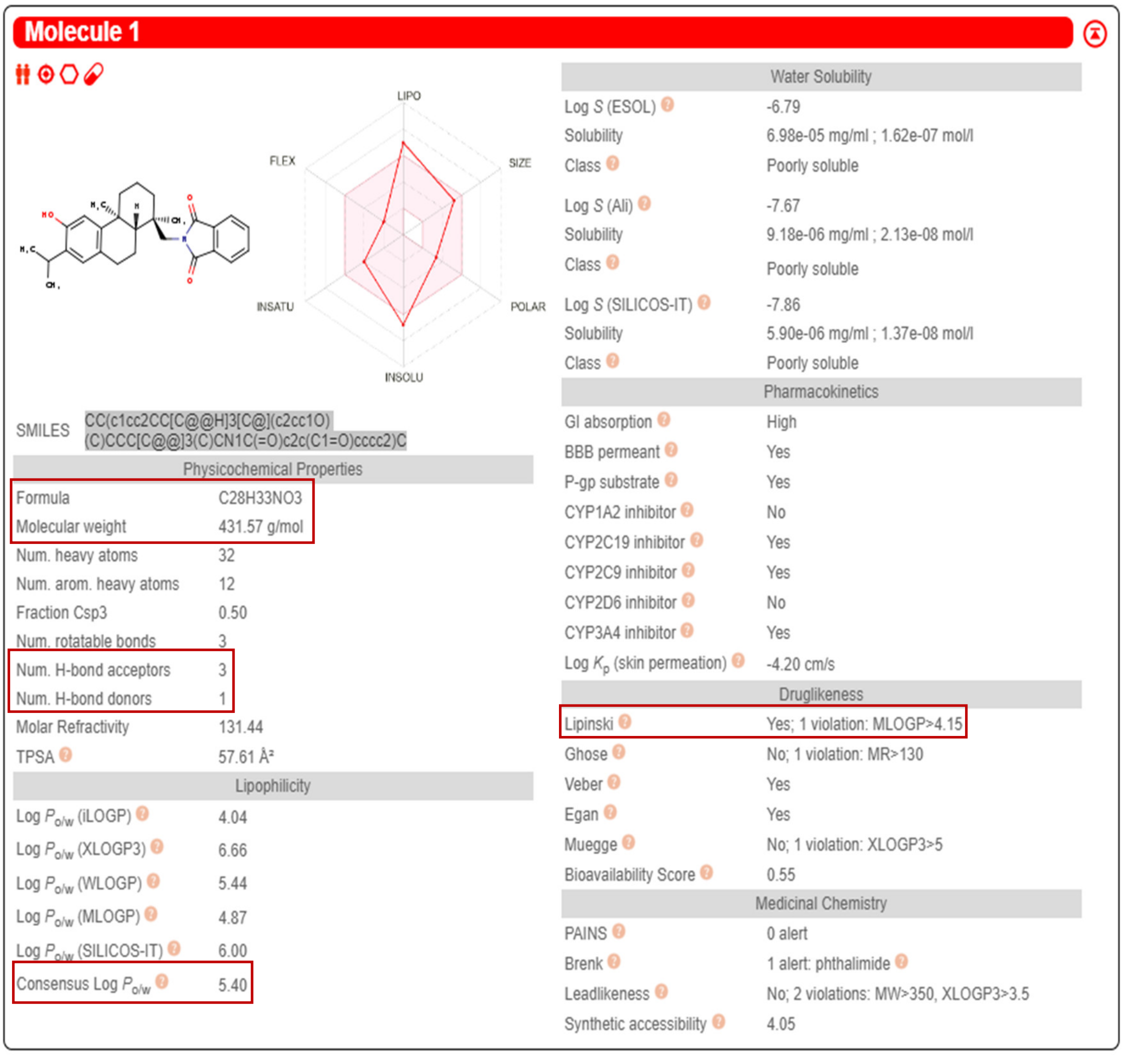

SMILES: CC(c1cc2CC[C@@H]3[C@](c2cc1O)(C)CCC[C@@]3(C)CN1C(=O)c2c(C1=O)cccc2)C

Molecule 1

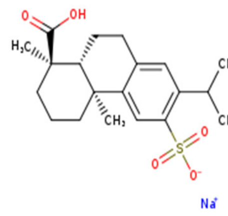

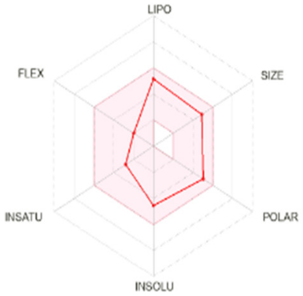

SMILES OC(=O)[C@]1(C)CCC[C@]2([C@H]1CCc1c2cc(c(c1)C(C)C)S(=O)(=O)[O-])C.[Na+]

Physicochemical Properties	
Formula	C20H27NaO5S
Molecular weight	402.48 g/mol
Num. heavy atoms	27
Num. arom. heavy atoms	6
Fraction Csp3	0.65
Num. rotatable bonds	3
Num. H-bond acceptors	5
Num. H-bond donors	1
Molar Refractivity	99.39
TPSA	102.88 Å²

Lipophilicity	
Log $P_{o/w}$ (iLOGP)	-11.29
Log $P_{o/w}$ (XLOGP3)	3.55
Log $P_{o/w}$ (WLOGP)	4.89
Log $P_{o/w}$ (MLOGP)	3.61
Log $P_{o/w}$ (SILICOS-IT)	3.27
Consensus Log $P_{o/w}$	0.81

Water Solubility	
Log *S* (ESOL)	-4.54
Solubility	1.17e-02 mg/ml ; 2.90e-05 mol/l
Class	Moderately soluble
Log *S* (Ali)	-5.40
Solubility	1.62e-03 mg/ml ; 4.02e-06 mol/l
Class	Moderately soluble
Log *S* (SILICOS-IT)	-4.62
Solubility	9.61e-03 mg/ml ; 2.39e-05 mol/l
Class	Moderately soluble

Pharmacokinetics	
GI absorption	High
BBB permeant	No
P-gp substrate	Yes
CYP1A2 inhibitor	No
CYP2C19 inhibitor	No
CYP2C9 inhibitor	No
CYP2D6 inhibitor	No
CYP3A4 inhibitor	No
Log K_p (skin permeation)	-6.23 cm/s

Druglikeness	
Lipinski	Yes; 0 violation
Ghose	Yes
Veber	Yes
Egan	Yes
Muegge	Yes
Bioavailability Score	0.56

Medicinal Chemistry	
PAINS	0 alert
Brenk	1 alert: sulfonic_acid_2
Leadlikeness	No; 2 violations: MW>350, XLOGP3>3.5
Synthetic accessibility	4.24

SMILES: OC(=O)[C@]1(C)CCC[C@]2([C@H]1CCc1c2cc(c(c1)C(C)C)S(=O)(=O)[O-])C.[Na+]

Table S1. In vitro pharmacokinetic parameters for compound phthFGL (**1a**): Caco2 permeability.[a]

	Caco2 Permeability				
Compound ID	AVG Papp A/B (nm/s)	SD Papp A/B	AVG Papp B/A (nm/s)	SD Papp B/A	Efflux Ratio (B2A/A2B)
Cambamazepine	718.9	12.5	565.9	32.8	0.8
Digoxin	46.1	4.6	270.6	20.5	5.9
**1a**	227.8	49.9	92.7	28.8	0.4

[a] See experimental methods in reference: Ling, T.; Tran, M.; González, M.A.; Gautam, L.N.; Connelly, M.; Wood, R.K.; Fatima, I.; Miranda-Carboni, G.; Rivas, F. (+)-Dehydroabietylamine derivatives target triple-negative breast cancer. *Eur. J. Med. Chem.* **2015**, *102*, 9–13. https://doi.org/10.1016/j.ejmech.2015.07.034

Table S2. In vitro pharmacokinetic parameters for compound phthFGL (**1a**): stability in microsomes and plasma.[a]

	Metabolic Stability (Human)			Metabolic Stability (Mouse)				Plasma Stability (Human)		Plasma Stability (Mouse)	
Compound ID	t1/2 (hr)	STD	Clint (mL/Min/Kg)	t1/2 (hr)	STD	Clint (mL/Min/Kg)		t1/2 (hr)	STD	t1/2 (hr)	STD
Verapermil	2.10	0.2	9.9	1.32	0.1	43.4	Eucatropine	0.57	0.01	4.96	0.28
1a	3.53	1.1	5.9	3.83	0.8	14.9	1a	52.58	6.11	>>48	--

[a] See experimental methods in reference: Ling, T.; Tran, M.; González, M.A.; Gautam, L.N.; Connelly, M.; Wood, R.K.; Fatima, I.; Miranda-Carboni, G.; Rivas, F. (+)-Dehydroabietylamine derivatives target triple-negative breast cancer. *Eur. J. Med. Chem.* **2015**, *102*, 9–13. https://doi.org/10.1016/j.ejmech.2015.07.034
